# Unilateral biportal endoscopic lumbar interbody fusion versus minimally invasive transforaminal lumbar interbody fusion in the treatment of lumbar degenerative diseases: a retrospective multicenter cohort study

**DOI:** 10.1371/journal.pone.0333165

**Published:** 2025-09-26

**Authors:** Xiang Chen, Jingbo Xie, Zhihui Zhang, Yi Liu, Liang Shi, Lang Hu

**Affiliations:** 1 Department of Spinal Orthopaedics, Fengcheng People’s Hospital, Fengcheng City, Jiangxi Province, China; 2 Department of Orthopedics, Xiangyang No.1 People’s Hospital, Hubei University of Medicine, Xiangyang City, Hubei Province, China; Nippon Medical School, JAPAN

## Abstract

**Background:**

Minimally invasive transforaminal lumbar interbody fusion (Mis-TLIF) is one of the most commonly used methods for lumbar fusion. However, in recent years, the unilateral biportal endoscopic lumbar interbody fusion (UBE-LIF) has also gradually attracted the attention of spine surgeons. This study aims to compare the perioperative and long-term clinical outcomes of the two procedures for lumbar degenerative diseases (LDD).

**Methods:**

We collected clinical data of patients who had undergone minimally invasive transforaminal lumbar interbody fusion (Mis-TLIF) or unilateral biportal endoscopic lumbar interbody fusion (UBE-LIF) for lumbar degenerative diseases (LDD) from January 2019 to December 2022. The primary outcome measure was the Oswestry Disability Index (ODI) at 12 months postoperatively. Secondary outcome measures included 12-month visual analog scale (VAS) scores for low back pain (LBP) and leg pain (LP), and postoperative complication rate.

**Results:**

There were no significant differences in the preoperative VAS scores for LBP, LP, or ODI between the two groups. The VAS score for LBP was significantly lower in the UBE-LIF group than in the Mis-TLIF group 1 week postoperatively (1.4 ± 1.1 vs. 2.1 ± 1.0, P = 0.001). However, there was no significant difference in the VAS scores for LBP, LP, and ODI at 1, 6, and 12 months postoperatively. The length of stay was significantly lower in the UBE-LIF than in the Mis-TLIF group (5.2 ± 1.1 vs. 6.3 ± 1.2 days, P < 0.001). The operative time (188.9 ± 19.8 vs. 159.5 ± 11.6 minutes, P < 0.001) of the UBE-LIF group was significantly higher than that of the Mis-TLIF group, while the estimated blood loss (131.0 ± 21.9 vs. 191.7 ± 23.3 ml, P < 0.001) and postoperative drainage volume (123.0 ± 55.4 vs. 191.2 ± 47.5 ml, P < 0.001) were significantly lower in the UBE-LIF than in the Mis-TLIF group. The complication rate was slightly higher in the UBE-LIF than in the Mis-TLIF group; however, the difference was not significant (11.5% vs. 5.0%, P = 0.299).

**Conclusion:**

UBE-LIF can achieve better perioperative clinical outcomes than Mis-TLIF. However, in the long-term, these two procedures can achieve equivalent clinical efficacy.

## Background

Lumbar degenerative diseases (LDD) cause significant pain and economic burden to patients worldwide [1–2]. According to Ravindra et al., LDD and low back pain (LBP) occur in approximately 266 million individuals (3.63%) worldwide [1]. In the United States, the average 2-year total cost of medical management (direct plus indirect) was $6,606 for lumbar spondylolisthesis, $7,747 for lumbar spinal stenosis, and $7,097 for lumbar disc herniation [2]. Spinal fusion surgery is widely used for the treatment of LDD. The incidence and cost of spinal fusion surgery for LDD have increased in recent years [3–5].

Minimally invasive transforaminal lumbar interbody fusion (Mis-TLIF), first proposed by Foley et al. [6], has been widely adopted for the surgical treatment of LDD because of its equivalent clinical efficacy, minimal trauma, and rapid recovery compared to conventional open procedures [7–11]. With the rapid development of endoscopic technology, endoscopic fusion has gradually been used for the clinical treatment of LDD [12]. Unilateral biportal endoscopic lumbar interbody fusion (UBE-LIF), an endoscopy-based fusion procedure first proposed by Heo et al. [13], is currently receiving increasing attention from spine surgeons [14,15].

Although MIS-TLIF is a decompression and fusion procedure performed under a tube by air medium. Compared with open surgery, it has less surgical trauma and faster recovery rate. However, UBE-LIF achieves wide operating space and precise decompression through dual-channel endoscopy mediated by water medium. In addition, the UBE procedure may have less surgical side effects [16–18]. Previous studies have compared the clinical efficacy of Mis-TLIF with UBE-TLIF [16–24]. However, there is still controversy regarding the advantages and disadvantages of these two procedures. In addition, previous studies reported only a limited number of cases. In this study, we aimed to compare the clinical outcomes and complications of Mis-TLIF and UBE-LIF for the treatment of LDD to further evaluate the advantages and disadvantages of these two procedures.

## Methods

This study retrospectively analyzed the collected clinical data of patients from two spine centers who underwent Mis-TLIF or UBE-LIF for LDD between January 2019 and December 2022. The data were accessed for research purposes between May 2024 and September 2024. This study was reviewed and approved by the hospital’s ethics committee. All patients provided written informed consent. The authors had access to information that could identify individual participants during or after data collection.

### Inclusion criteria

1). LDD patients aged 40–80 years. 2). Patients who underwent Mis-TLIF or UBE-LIF 3). Primary lumbar spinal surgery.

### Exclusion criteria

1). Patients undergoing revision lumbar surgery. 2). With severe spinal deformity requiring deformity correction surgery. 3). Lack of clinical or imaging data. 4). Severe underlying diseases, including myocardial infarction, cerebral infarction, severe pulmonary disease, and Parkinsonism. 5). Patients who were followed up for less than one year or were lost to follow-up. 6). Patients who refused to participate.

### Collection of demographics

An independent person completed the collection of basic patient information and follow-up. Basic patient information included sex, age, body mass index (BMI), types of comorbidities, diagnosis and surgical methods, and length of stay (LOS).

### Patient allocation

The allocation of patients in this study was not random, but was based on operator preferences and the time order in which the surgeon performs the surgery (Mis-TLIF are performed earlier in both spine centers).

### Surgical method

Both Mis-TLIF and UBE-LIF were performed by two spine surgeons (XC and LH) with 5 years of experience in spine surgery from the two spine centers, respectively. The detailed surgical procedure is described below.

#### Mis-TLIF.

The procedure of Mis-TLIF was performed according to the method reported by Foley [6]. After general anesthesia and intubation, the patient was placed in the prone position, C-arm X-ray fluoroscopy was used to locate the target pedicle and intervertebral space, and routine disinfection and draping were performed. A straight incision, 3 cm beside the spinous process of the target intervertebral space was made, and the skin, subcutaneous tissue, and lumbodorsal fascia were incised layer-by-layer. An expandable tube was placed through the multifidus and longissimus muscle spaces to expand step-by-step, and a cold light source was connected to fully expose the articular process and lamina. The inferior articular process of the upper vertebral body, the superior articular process of the lower vertebral body, and part of the vertebral lamina were resected using gun forceps under direct vision. The hypertrophic ligamentum flavum was resected; the nerve root canal was fully decompressed and enlarged; the dura mater and walking root were exposed; the diseased intervertebral disc was resected; and the compressed walking nerve root was removed. The endplate was then thoroughly treated, the autogenous bone and interbody fusion cage were implanted in the intervertebral space, and the position of the fusion cage was confirmed by C-arm X-ray fluoroscopy. On the side of the incision, two pedicle screws were placed through the multifidus muscle space and fixed with a connecting rod. Subsequently, the contralateral skin and fascia were incised, the pedicle screw was placed under fluoroscopic guidance, a connecting rod was installed, and the top wire was placed to fix the surgical segment. A section of negative-pressure drainage was placed, and the wound was closed using layer-by-layer suturing.

#### UBE-LIF.

After intubation under general anesthesia, the patient was placed in a prone position, and segmental confirmation was performed under the guidance of C-arm X-ray fluoroscopy. A positioning needle was inserted into the paraspinal muscle at the center of the desired surgical segment to confirm that it was at the center of the target level. The surgical site was routinely disinfected and draped. The two incisions were 1 cm above and 1 cm below the center of the positioning needle. The water pressure of the saline infusion pump was maintained below 30 mmHg, the endoscope was placed in the observation tube, and the surgical instruments were placed in the working tube. Partial ipsilateral laminectomy, followed by partial facet resection, was performed arthroscopically using Kirschner forceps and an osteotome. Following partial ipsilateral laminectomy and facet resection, contralateral sublaminar decompression was performed. The bone particles obtained during surgery were used for intervertebral bone grafting. After the resection of the ipsilateral facet joint, a passage into the intervertebral foramen was created. The ligamentum flavum covering the dura mater and the outlet nerve root was resected after decompression of the ipsilateral and contralateral bony stenoses. The disc was dissected using a radiofrequency probe in the foraminal space between the exit and the walking roots, and discectomy was performed using nuclear forceps and a curette. After the arthroscope entered the intervertebral space, the cartilage endplate was processed, and the intervertebral space directly viewed under a magnified arthroscope to expose the subchondral bone. During interbody bone grafting, a special catheter was used to prevent continuous lavage until the final bone loss. After bone grafting was completed, the interbody cage was placed under the protection of two small retractors for the exit and walking roots, and the position of the fusion cage was confirmed using C-arm X-ray fluoroscopy. The contralateral skin and fascia were then incised, and pedicle screws were placed under fluoroscopic guidance. Two ipsilateral percutaneous pedicle screws were placed through the two previously used portals to install the connecting rod and fix the surgical segment. Negative pressure drainage was performed, and the wound was closed using layer-by-layer suturing.

### Discharge

Patients were discharged after removal of the drainage tube and completion of a lumbar radiographic examination, with a visual analog scale (VAS) score for low back pain (LBP) or leg pain (LP) below 3 and no evidence of poor wound healing.

### Follow-up

An independent clinician recorded operative time (OT), estimated blood loss (EBL), postoperative drainage volume (PDV), and length of hospital stay (LOS). EBL was calculated by subtracting the volume of intraoperative irrigation fluid from the volume of blood in the collection container at the end of the procedure and subtracting the volume of blood in the preoperative collection container by the circulating nurse‌. In addition, VAS scores for LBP and LP were assessed and recorded preoperatively and at 1 week, 1 month, 6 months, and 12 months postoperatively by independent follow-up personnel. In addition, the Oswestry Disability Index (ODI) was assessed and recorded before surgery and 1, 6, and 12 months postoperatively.

### Assessment of surgical complications

The surgical complications were assessed by an operating surgeon. Surgical complications included iatrogenic dural tears, epidural hematomas, surgical site infections, poor wound healing, and nerve root injury, etc.

### Statistical methods

The Kolmogorov–Smirnov test was used to test whether continuous data conformed to a normal distribution. For continuous variables, the student’s t-test (continuous normally distributed variables) or Mann–Whitney U test (non-normally distributed variables) was used for the analysis. For categorical variables, the chi-squared test or fisher’s exact test was used. Statistical significance was set at P < 0.05. SPSS.26 software (IBM Corp., Armonk, New York, USA) was used for data analysis.

## Results

### Inclusion and exclusion

**Fig 1:**
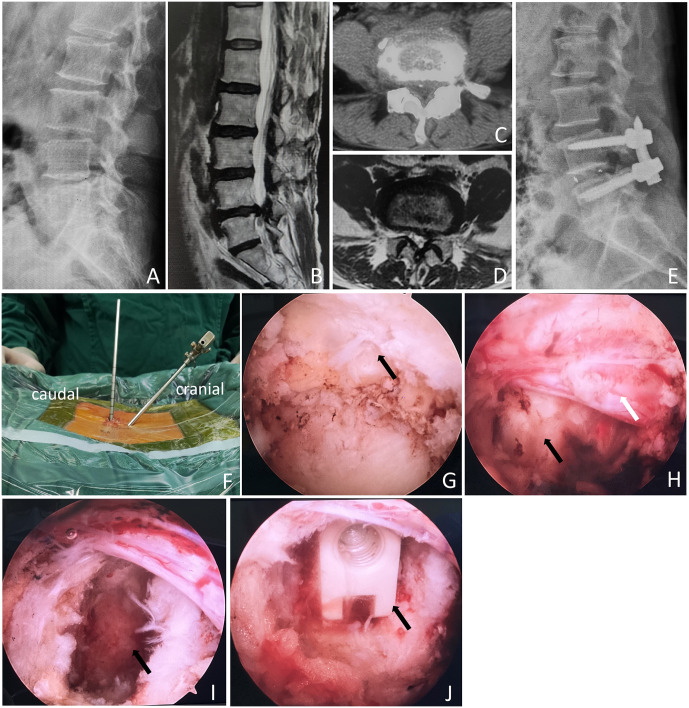
A patient with lumbar disc herniation (LDH) accompanied by spinal stenosis who underwent UBE-LIF. A showed the preoperative lumbar lateral X-ray. B showed LDH with spinal stenosis on the L4/5 in preoperative lumbar sagittal MRI. C and D showed the presence of LDH with spinal stenosis on the L4/5 axis, respectively. E showed the internal fixation after UBE-LIF was in good position after surgery. F showed the observation tube (cranial) and working tube (caudal) of UBE-LIF. G showed the endoscopic removal of the ligamentum flavum (black arrow). H showed endoscopic exposure of the nerve root (white arrow) and herniated disc (black arrow). I showed the endoscopic removal of intervertebral discs (black arrow). J showed the cage (black arrow) was implanted endoscopically.

**Fig 2:**
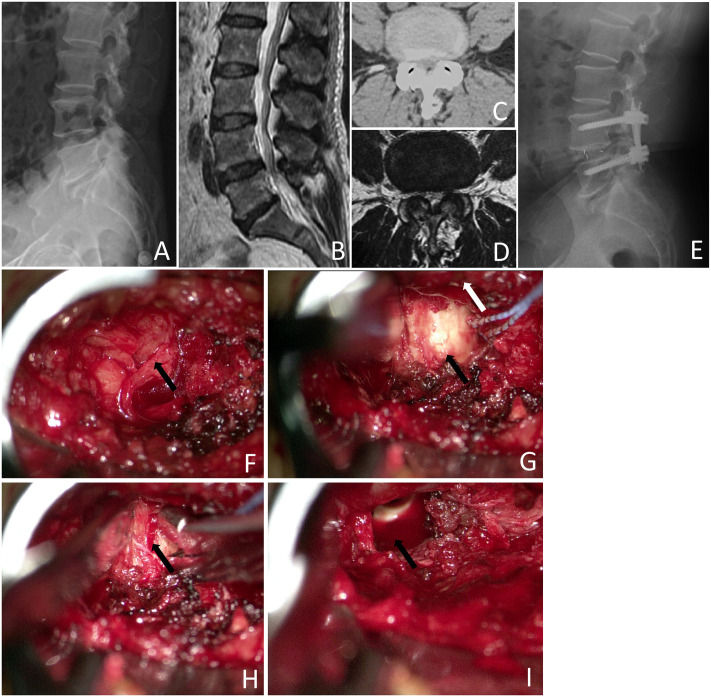
A patient with lumbar spinal stenosis (LSS) who underwent Mis-TLIF. A showed the preoperative lumbar lateral X-ray. B showed LSS on the L4/5 in preoperative lumbar sagittal MRI. C and D showed the presence of LSS on the L4/5 axis, respectively. E showed the internal fixation after Mis-TLIF was in good position after surgery. F showed the microscopic removal of the ligamentum flavum (black arrow). G showed microscopic exposure of the nerve root (white arrow) and intervertebral discs (black arrow). H showed the microscopic removal of intervertebral discs (black arrow). I showed that cage (black arrow) implanted microscopically.

**Fig 3:**
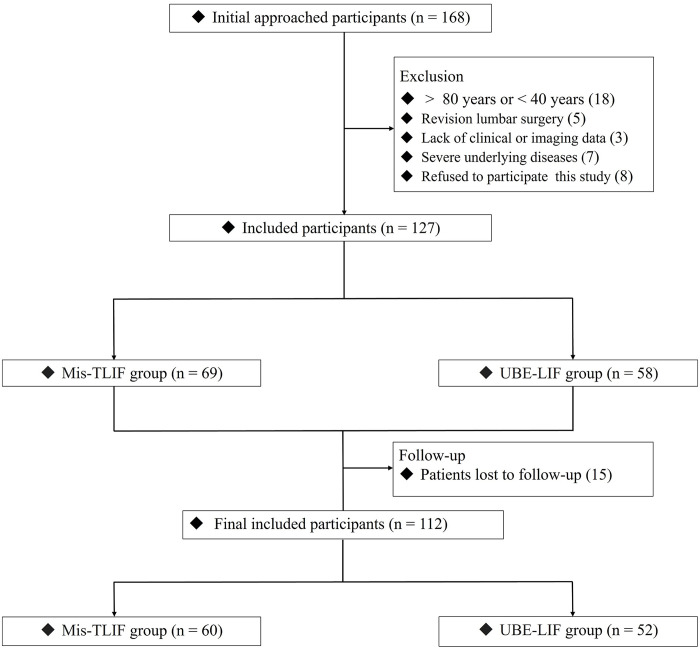
The inclusion and exclusion flowchart of this study.

The inclusions and exclusions are illustrated in Fig. 1. Initially, 168 patients were included in the study. After excluding 12 patients younger than 40 years, 5 patients older than 80 years, 6 patients who underwent revision surgery, 4 patients who lacked clinical and imaging data, 3 patients with severe underlying diseases, and 12 patients who refused to participate in the study, 127 patients were included. After excluding 15 patients who were lost to follow-up, 112 patients were included in this study: 60 in the Mis-TLIF group and 52 in the UBE-LIF group. Based on operator preferences and the time order in which the surgeon performs the surgery, the MIS-TLIF procedure was performed in most patients (56 cases of MIS-TLIF, 5 cases of UBE-LIF) from January 2019 to August 2021. From September 2021 to December 2022, most patients (47 cases of UBE-LIF, 4 cases of MIS-TLIF) in this study underwent UBE-TLIF surgery. The Figs. 2 and 3 showed a typical case underwent MIS-TLIF and UBE-LIF surgery, respectively.

### Demographics

There were no significant differences in sex, age, BMI or preoperative comorbidities between the two groups. The LOS was significantly lower in the UBE-LIF group than in the Mis-TLIF group (5.2 ± 1.1 vs. 6.3 ± 1.2 days, P < 0.001) (Table 1).

### Clinical outcomes

**Fig 4:**
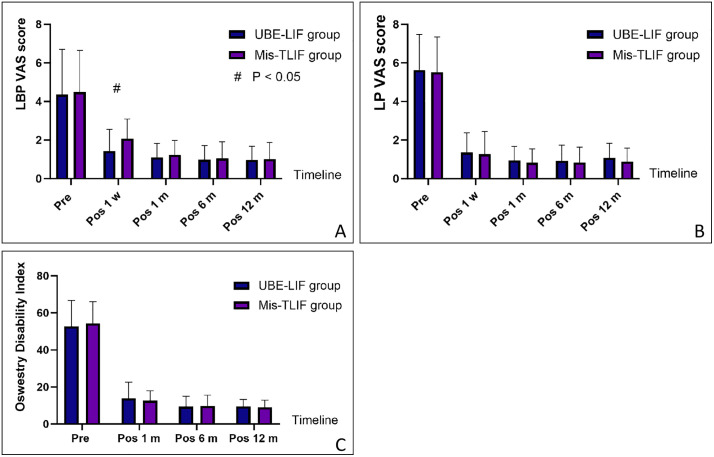
The VAS scores for LBP and LP and ODI of the two groups. A: The VAS scores for LBP in the UBE-LIF group were significantly lower than those in the Mis-TLIF group 1 week after surgery. B: There was no significant difference in VAS scores for LP between the two groups. C: There was no significant difference in ODI between the two groups.

Fig 4 and Table 2 showed the clinical outcomes of the two groups. There were no significant differences in the preoperative VAS score and ODI for LBP or LP between the two groups. The VAS score for LBP was significantly lower in the UBE-LIF than in the Mis-TLIF group 1 week after surgery (1.4 ± 1.1 vs. 2.1 ± 1.0, P = 0.001). However, there were no significant differences in the VAS scores for LBP, LP, and ODI at 1, 6, and 12 months after surgery.

**Table 1:** Comparison of demographics of the two groups.

Subgroup	Mis-TLIF group (60)	UBE-LIF group (52)	P value
Sex (man/woman)	31/29	25/27	0.705
Age (year)	62.3 ± 6.3	60.3 ± 7.8	0.142
BMI (kg/m2)	25.8 ± 1.8	25.8 ± 1.7	0.880
Comorbidities	14(23.3%)	11(21.2%)	0.782
OT (minute)	159.5 ± 11.6	188.9 ± 19.8	< 0.001
EBL (ml)	191.7 ± 23.3	131.0 ± 21.9	< 0.001
PDV (ml)	191.2 ± 47.5	123.0 ± 55.4	< 0.001
LOS (day)	6.3 ± 1.2	5.2 ± 1.1	< 0.001

BMI, body mass index; OT, operative time; EBL, estimated blood loss; PDV, postoperative drainage volume; LOS, length of stay.

**Table 2:** Comparison of LBP, LP, ODI of the two groups.

Subgroup	Mis-TLIF group (60)	UBE-LIF group (52)	P value
Pre LPB VAS	4.5 ± 2.2	4.4 ± 2.3	0.817
Post LBP VAS (1 w)	2.1 ± 1.0	1.4 ± 1.1	0.001
Post LBP VAS (1 m)	1.2 ± 0.7	1.1 ± 0.7	0.326
Post LBP VAS (6 m)	1.1 ± 0.9	1.0 ± 0.7	0.647
Post LBP VAS (12 m)	1.0 ± 0.9	1.0 ± 0.7	0.813
Pre LP VAS	5.5 ± 1.8	5.6 ± 1.8	0.735
Post LP VAS (1 w)	0.8 ± 0.7	0.9 ± 0.7	0.638
Post LP VAS (1 m)	0.8 ± 0.8	0.9 ± 0.8	0.362
Post LP VAS (6 m)	0.8 ± 0.8	0.8 ± 0.8	0.560
Post LP VAS (12 m)	0.9 ± 0.7	1.1 ± 0.8	0.138
Pre ODI	54.2 ± 11.9	52.7 ± 14.0	0.539
Post ODI (1 m)	12.6 ± 5.3	13.8 ± 8.7	0.377
Post ODI (6 m)	9.6 ± 6.0	9.4 ± 5.6	0.780
Post ODI (12 m)	9.2 ± 3.7	9.5 ± 3.8	0.629

LBP, low back pain; LP, Leg pain; VAS, visual analog scale; ODI, oswestry disability index.

**Table 3:** Comparison of surgical complications of the two groups.

Subgroup	Mis-TLIF group (60)	UBE-LIF group (52)	P value
Complication rate	5.0%	11.5%	0.299
Iatrogenic dura tear	1	3	
Transient nerve injury	1	1	
Ganglionitis	0	1	
Poor wound healing	1	0	
Epidural hematoma	0	1	

### Surgical-related outcomes

The OT of the UBE-LIF group was significantly higher than that of the Mis-TLIF group (188.9 ± 19.8 vs. 159.5 ± 11.6 minutes, P < 0.001), while the EBL (131.0 ± 21.9 vs. 191.7 ± 23.3 ml, P < 0.001) and PDV (123.0 ± 55.4 vs. 191.2 ± 47.5 ml, P < 0.001) of the UBE-LIF group were significantly lower than those of the Mis-TLIF group (Table 1).

### Surgical complications and management

Overall, the surgical complication rate was slightly higher in the UBE-LIF than in the mis-TLIF group; however, this difference was not significant (11.5% vs. 5.0%, P = 0.299) (Table 3). In the Mis-TLIF group, one patient had an iatrogenic dural tear without additional repair. The patient developed postoperative cerebrospinal fluid leakage. One patient had decreased muscle strength and numbness in the lower extremities postoperatively, but muscle strength was restored one week after surgery; therefore, he was classified as having a transient nerve injury. One patient experienced poor wound healing and underwent debridement under local anesthesia. In the UBE-LIF group, three patients had iatrogenic dural tears without additional repair. Transient nerve injury also occurred in one patient as he experienced decreased muscle strength and numbness for 3 weeks after surgery. Ganglionitis occurred postoperatively in one patient whose pain was relieved by conservative treatment (2 weeks of oral celecoxib and 3 weeks of pregabalin). One patient was diagnosed with suspected epidural hematoma (by magnetic resonance imaging) after surgery because of referred pain in the lower extremity but was treated conservatively (intravenous mannitol and steroids for 1 week).

## Discussion

This study compared the perioperative and long-term clinical outcomes of the two LDD procedures. The VAS score for LBP was significantly lower in the UBE-LIF than that in the MIS-TLIF group 1 week after surgery. However, there were no significant differences in the VAS scores for LBP, LP, and ODI at 1, 6, and 12 months after surgery. The LOS was significantly lower in the UBE-LIF group than that in the Mis-TLIF group. The OT in the UBE-LIF group was significantly higher than that in the Mis-TLIF group, whereas the EBL and PDV were significantly lower in the UBE-LIF group than those in the Mis-TLIF group.

The superiority and inferiority of UBE-LIF and Mis-TLIF remains controversial [16–24]. In 2021, Kang et al. reported the clinical and radiological outcomes of UBE-LIF (47 patients) and Mis-TLIF (32 patients) in patients with single- or two-segment lumbar spinal stenosis with or without spondylolisthesis. They found that the VAS scores for LBP and SF-36 scores were significantly improved in the UBE-LIF group compared to the Mis-TLIF group at 1 month postoperatively. However, there were no significant differences between the groups in the mean VAS scores for LBP and LP, ODI, and SF-36 scores at 1 year postoperatively [16]. Additionally, although the total OT was significantly longer in the UBE-LIF group, the EBL and PDV were significantly higher in Mis-TLIF group (P < 0.001). There were no between-group differences in fusion and postoperative complication rates. Kim et al. compared the clinical outcomes of UBE-LIF (32 patients) and Mis-TLIF (55 patients) for single-level isthmic or degenerative spondylolisthesis. They found that the VAS scores for LBP at 2 weeks and 2 months postoperatively were significantly lower in the UBE-LIF group (P = 0.001). Additionally, the difference in the fusion rates between the UBE-LIF and Mis-TLIF groups was not significant (93.7% vs. 92.7%, P = 0.43) [17]. Huang et al. enrolled 103 eligible patients with LDD who underwent UBE-LIF (n = 46) and Mis-TLIF (n = 57). They found that C-reactive protein and creatine kinase levels were generally lower in the UBE-LIF than in the Mis-TLIF group, especially the C-reactive protein levels on 1 and 3 days and creatinine kinase levels on 1 day postoperatively. True total blood loss, PDV, and hidden blood loss were significantly lower in the UBE-LIF than in the Mis-TLIF group. Postoperative LOS was statistically significantly shorter in the UBE-LIF group. Additionally, they found that VAS scores for LBP were significantly lower in the UBE-LIF group at 3 days and 1 month [20]. Arunakul et al. compared the clinical outcomes of UBE-LIF and Mis-TLIF for the treatment of LDD. They found that the UBE-LIF group (34 patients) showed a more substantial improvement in the VAS scores for LBP at 3 weeks postoperatively than the Mis-TLIF group (56 patients) [22]. Additionally, the study by Zhu et al. also found that the VAS score for LBP was significantly lower in the UBE-LIF group than in the MIS-TLIF group at 2 weeks and 1 month postoperatively [24]. In the present study, we found results similar to those of a previous study. The VAS score for LBP was significantly lower in the UBE-LIF than in the Mis-TLIF group 1 week after surgery (1.4 ± 1.1 vs. 2.1 ± 1.0, P = 0.001). However, there were no significant differences in the VAS scores for LBP, LP, and ODI at 1, 6, and 12 months after surgery. This is not difficult to explain because UBE-LIF is less damaging to the lumbar muscles than Mis-TLIF; in particular, Mis-TLIF requires retraction of the multifidus muscles using a retractor. Unlike Mis-TLIF, UBE-LIF does not involve the placement of a tubular retractor between the paraspinal muscles, thereby reducing direct ischemic injury [25,26]. However, the early differences of VAS score for LBP diminish over time. This is not difficult to explain, because as patients recover after surgery, the effect of UBE-LIF on more soft-tissue preservation and less muscle damage than MIS-TLIF is gradually diluted.

Additionally, although we found that the OT of the UBE-LIF group (188.9 ± 19.8 vs. 159.5 ± 11.6 minutes, P < 0.001) was significantly higher than that of the Mis-TLIF group, the EBL (131.0 ± 21.9 vs. 191.7 ± 23.3 ml, P < 0.001) and PDV (123.0 ± 55.4 vs. 191.2 ± 47.5 ml, P < 0.001) were significantly lower. A possible reason for this is that UBE-LIF is completed in a water medium, whereas mis-TLIF is completed in an air medium. In a water environment, intraoperative bleeding can be significantly reduced. There was less postoperative drainage in the UBE-LIF group, which again demonstrated less surgical trauma and a better hemostatic effect of this surgical method. The LOS was significantly lower in the UBE-LIF group than in the Mis-TLIF group (5.2 ± 1.1 vs. 6.3 ± 1.2 days, P < 0.001). We believe that the shorter LOS of the UBE-LIF group is also related to its lower PDV compared to that of the Mis-TLIF group.

Previous studies have not found a significant difference in complication rates between the two surgical procedures [17,20,22]. Kim et al. reported similar complication rates for UBE-LIF and Mis-TLIF (6.3% vs. 5.5%, P = 1.000). In the UBE-LIF group, one case of postoperative epidural hematoma and one case of transient palsy occurred, while in the Mis-TLIF group, one case of postoperative epidural hematoma and two cases of transient palsy occurred [17]. Huang et al. also reported similar complication rates for UBE-LIF and Mis-TLIF (5.2% vs. 4.5%, P = 0.489), including two small dural tears in the UBE-LIF group and two cases of transient ipsilateral dysesthesia in the Mis-TLIF group [20]. Arunakul et al. reported low complication rates in both groups (Mis-TLIF, 3.56% vs. UBE-LIF, 2.94%), with one case of hematoma and one of surgical site infection in the Mis-TLIF group, and one case of incomplete decompression in the UBE-LIF group [22]. However, in this study, the complication rate in the UBE-LIF group was slightly higher than that in the Mis-TLIF group, although the difference was not significant (11.5% vs. 5.0%, P = 0.299). This may be due to the steeper learning curve of UBE-LIF because the mean OT of UBE-LIF was also significantly higher than that of Mis-TLIF. However, after overcoming the learning curve of UBE-LIF, we believe that this procedure may become an alternative to Mis-TLIF. Additionally, spine surgeons could effectively overcome the learning curve of UBE-LIF, such as structured training programs, cadaveric workshops and unilateral biportal endoscopic decompression surgery.

## Limitations

This study has several limitations. First, this study was not a randomized controlled trial. The assignment of patients to surgery was not random but was based on operator preferences and time order in which the surgeon performs the surgery (Mis-TLIF is performed earlier in both spine centers), which may have resulted in bias in case selection. For example, due to earlier perform and mastery of Mis-TLIF surgery, surgeons may tend to choose Mis-TLIF for some complex cases. However, it should be noted that the surgeries included in this study were performed by two surgeons with 5 years of experience in spinal surgery, which may have reduced the interference of non -random patient allocation on the conclusions to some extent. Additionally, although the patient allocation in this study was not random, i believe that the patients allocation based on time order in which the surgeon performs the surgery also reduces the bias caused by case selection to some extent. Because during a certain period of time, most of the surgeries performed by the surgeon are consistent. In addition, despite the calculation of the sample size, the total number of cases in this study was limited. In addition, the follow-up period for this study was only 12 months; a longer follow-up period is warranted to further clarify the advantages and disadvantages of these two procedures. In addition, the patient-reported outcomes (PROs) in this study included only VAS and ODI scores. Other PROs, such as the 36-Item Short Form Health Survey and EuroQol five-dimensional questionnaire, were not adopted. Besides, despite reporting clinical outcomes, this study lacks complete radiological outcomes, such as fusion rates, subsidence, or adjacent segment degeneration. Finally, the results of this study were obtained from only two spinal centers, and more spinal center data are needed to validate this conclusion.

## Conclusion

This study compared the perioperative and long-term clinical outcomes of UBE-LIF and Mis-TLIF for LDD. In general, we found that UBE-LIF achieved better perioperative clinical outcomes than Mis-TLIF. However, in the long-term, these two procedures can achieve equivalent clinical efficacy.

## Supporting information

S1 Data Raw data. (XLSX)

## Abbreviations

BMI: body mass index
EBL: estimated blood loss
LBP: low back pain
LDD: lumbar degenerative diseases
LDH: lumbar disc hernation
LOS: length of stay
LP: leg pain
Mis-TLIF: minimally invasive transforaminal lumbar interbody fusion
ODI: Oswestry Disability Index
OT: operating time
PDV: postoperative drainage volume
PROs: patient-reported outcomes
UBE-LIF: unilateral biportal endoscopic lumbar interbody fusion
VAS: visual analog scale.
